# DNA from the Past Informs *Ex Situ* Conservation for the Future: An “Extinct” Species of Galápagos Tortoise Identified in Captivity

**DOI:** 10.1371/journal.pone.0008683

**Published:** 2010-01-13

**Authors:** Michael A. Russello, Nikos Poulakakis, James P. Gibbs, Washington Tapia, Edgar Benavides, Jeffrey R. Powell, Adalgisa Caccone

**Affiliations:** 1 Department of Biology, University of British Columbia Okanagan, Kelowna, British Columbia, Canada; 2 Department of Biology and Natural History Museum of Crete, University of Crete, Irakleio, Crete, Greece; 3 College of Environmental Sciences and Forestry, State University of New York, Syracuse, New York, United States of America; 4 Galápagos National Park Service, Puerto Ayora, Galápagos, Ecuador; 5 Department of Ecology and Evolutionary Biology, Yale University, New Haven, Connecticut, United States of America; University of Otago, New Zealand

## Abstract

**Background:**

Although not unusual to find captive relicts of species lost in the wild, rarely are presumed extinct species rediscovered outside of their native range. A recent study detected living descendents of an extinct Galápagos tortoise species (*Chelonoidis elephantopus*) once endemic to Floreana Island on the neighboring island of Isabela. This finding adds to the growing cryptic diversity detected among these species in the wild. There also exists a large number of Galápagos tortoises in captivity of ambiguous origin. The recently accumulated population-level haplotypic and genotypic data now available for *C. elephantopus* add a critical reference population to the existing database of 11 extant species for investigating the origin of captive individuals of unknown ancestry.

**Methodology/Findings:**

We reanalyzed mitochondrial DNA control region haplotypes and microsatellite genotypes of 156 captive individuals using an expanded reference database that included all extant Galápagos tortoise species as well as the extinct species from Floreana. Nine individuals (six females and three males) exhibited strong signatures of Floreana ancestry and a high probability of assignment to *C. elephantopus* as detected by Bayesian assignment and clustering analyses of empirical and simulated data. One male with high assignment probability to *C. elephantopus* based on microsatellite genotypic data also possessed a “Floreana-like” mitochondrial DNA haplotype.

**Significance:**

Historical DNA analysis of museum specimens has provided critical spatial and temporal components to ecological, evolutionary, taxonomic and conservation-related research, but rarely has it informed *ex situ* species recovery efforts. Here, the availability of population-level genotypic data from the extinct *C. elephantopus* enabled the identification of nine Galápagos tortoise individuals of substantial conservation value that were previously misassigned to extant species of varying conservation status. As all captive individuals of *C. elephantopus* ancestry currently reside at a centralized breeding facility on Santa Cruz, these findings permit breeding efforts to commence in support of the reestablishment of this extinct species to its native range.

## Introduction

The 2009 IUCN Red List includes 65 species of plants and animals that are officially extinct in the wild, many of which continue to persist in captivity [1]. These captive relicts of species lost from their native ranges are increasingly common, subject to intensive conservation management to prevent outright extinction [2].

**Figure 1:**
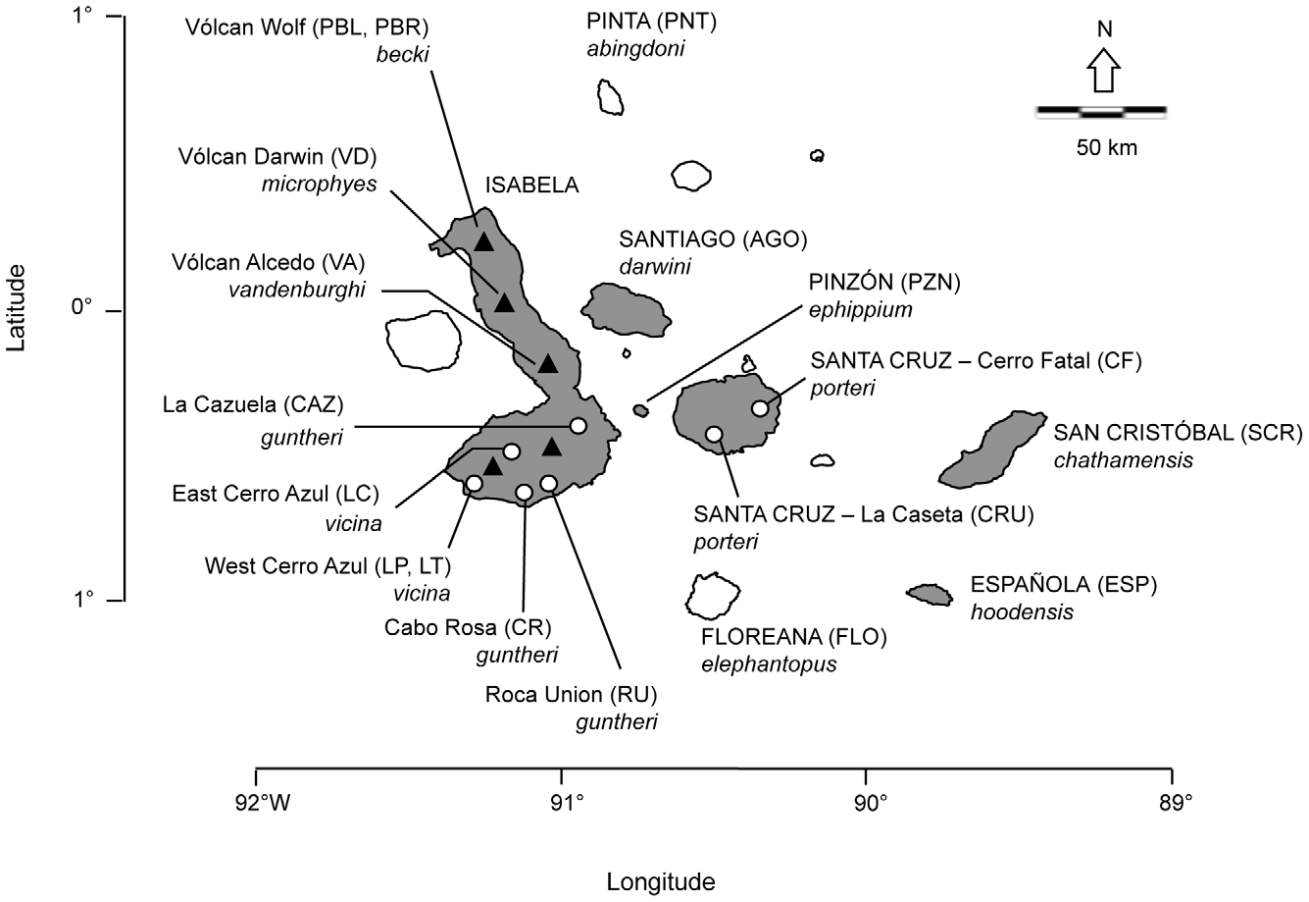
Distribution of giant tortoises throughout the Galápagos archipelago. Island names are capitalized; shaded islands indicate presence of extant tortoise populations (species names italicized). Populations are indicated by geographical name (e.g., Vólcan Wolf) and associated sampling site (e.g., PBL, PBR). Triangles represent volcanoes on Isabela Island and circles indicate additional sampling locations. Figure modified from Russello et al. [5].

The Galápagos tortoises represent a group of 11 extant species (*Chelonoidis spp.*; Figure 1, see Materials and Methods for description of recognized taxonomy), many of which are imperiled and the object of extensive *in situ* and *ex situ* conservation efforts ranging from control of poaching, protection of habitat, head-starting of *C. ephippium* on Pinzon Island, and captive breeding and repatriation of *C. hoodensis* to Española Island [3]. Previous genetic surveys investigating the origin of captive individuals of unknown ancestry provided managers critical historical information for maintaining the integrity of distinct lineages [4], [5]. These studies collectively examined 156 individuals of unknown ancestry held in captive populations on three continents, assigning them to the species level and, in many cases, to their population of origin [4], [5]. Not surprisingly, the majority of individuals assigned to tortoise populations on Santa Cruz and Isabela islands that are easily accessible and have been historically harvested. Fifteen individuals, however, were assigned to critically endangered species (e.g. *C. ephippium*, Pinzón Island) or to natural populations of known mixed ancestry (e.g. Volcano Wolf, Isabela Island)[4], [5].

Yet, the power of population assignment approaches is fundamentally linked to the underlying reference population database. If the population of origin of an individual is not represented in the sampled set of reference populations, the assignment algorithms will still designate a population of origin, albeit an incorrect one [6]. When additional reference population data become available either through expanded sampling across space (e.g. broader geographic coverage of contemporary distribution) or time (e.g. recently extinct species or extirpated populations), reanalysis of population assignments for individuals of unknown ancestry may be warranted. Such reanalyses may be particularly important when research questions have direct relevance to on-going conservation strategies.

It has been well-publicized [7] that the Pinta Island tortoise *C. abingdoni* is extinct in the wild (currently represented by a solitary male in captivity, Lonesome George), yet another species endemic to Floreana Island (*C. elephantopus*) was already extinct at the time of Van Denburgh's [8] taxonomic revision in the early 1900's. A recent study reported living descendents of the extinct *C. elephantopus* on the neighboring island of Isabela, and suggested that the removal of multiple individuals may aid in the establishment of a captive breeding program [9] and eventual reintroduction to Floreana. The population-level mitochondrial DNA (mtDNA) haplotypic and microsatellite genotypic data collected for *C. elephantopus* by way of historical DNA analysis of museum specimens [9] added a critical reference population to the existing database of extant species for investigating the origin of individuals of unknown ancestry.

In this study, we reanalyzed mtDNA haplotypic and microsatellite genotypic data for 156 captive individuals relative to the expanded reference population database that now includes the extinct *C. elephantopus* from Floreana to test hypotheses of ancestry set forth in Burns et al. [4] and Russello et al. [5]. Here we report the identification of individuals of recent Floreana ancestry that currently reside in a captive population in Galápagos. We further examined the relatedness of these individuals and discussed their utility for serving as a nucleus for re-establishing tortoises on Floreana Island that have now been absent for over a century.

## Results

Our sample of 156 captive individuals were assigned to their population(s) of origin based on mtDNA haplotypic and microsatellite genotypic data relative to reference databases including all extant species and the extinct species from Floreana (Table S1). As revealed in earlier studies by Burns et al. [4] and Russello et al. [5], all but two reanalyzed individuals possessed haplotypes originally sampled from species on Isabela (62.8%) or Santa Cruz (35.9%) Islands. Two individuals (PRZ01, CDRS037) exhibited haplotypes from Pinzon and San Cristóbal Islands, respectively. Interestingly, 13 individuals possessed northern Isabela haplotypes sampled at the Puerto Bravo and Piedras Blancas sites previously shown to cluster phylogenetically with haplotypes from other *Chelonoidis* species on Española, San Cristóbal and southern Isabela [10] as well as Floreana [9].

The genotypic assignment tests of Rannala and Mountain [11] and Pritchard et al. [12] exhibited a high degree of overlap, yielding consistent species assignments for 78.8% of the individuals sampled. Overall, the genotypic assignments corroborated the results obtained from the mtDNA analyses, with 129 individuals (82.6%) consistently assigned to the same locality by both datasets. The other 27 individuals exhibited patterns of mixed ancestry. Specifically, nine of 27 such individuals were alternatively assigned to different species on Isabela Island (Table S1). The remaining individuals were all assigned to the La Caseta *C. porteri* population on Santa Cruz or *C. becki* populations on Volcano Wolf by way of mtDNA, but assigned to different species according to their multi-locus genotypes (Table S1). The high degree of mixed ancestry detected was not surprising, as 36 individuals from the Santa Cruz breeding facility were sampled from a known “progeny” pen [4]. These individuals are direct descendents of founders from multiple Galápagos tortoise species that were housed together in a group enclosure [i.e. “parental” pen; 4] prior to knowledge of their origin and taxonomic assignment.

Of immediate interest, nine captive individuals exhibited congruent signatures of Floreana ancestry (Table 1), one of which (CDRS047) also possessed a “Floreana-like” mtDNA haplotype [haplotype 83; 9]. The remaining eight individuals with nuclear DNA assignment to Floreana, including the two females (CDRS106 & 107) currently housed with Lonesome George, possessed an “Española-like” haplotype only sampled in Puerto Bravo on northern Isabela Island [10]. The Puerto Bravo population hosts the living descendents of the near-extinct *C. abingdoni* (Pinta) and extinct *C. elephantopus* (Floreana) previously detected by Russello et al. [13] and Poulakakis et al. [9]. These findings were consistent with historical records and anecdotal accounts, as at least two of the nine individuals of Floreana ancestry (CDRS106, CDRS107) were originally captured from the wild population in Puerto Bravo, while no less than two additional females were collected from unspecified locations on Isabela in 1966 and subsequently housed in the parental pen (M. Castro, pers. com).

**Table 1:** Captive Galápagos tortoises of unknown origin with signatures of Floreana ancestry.

		Mitochondrial DNA control region				Microsatellite multi-locus genotypes								
						Pritchard et al. (2000)			Rannala & Mountain (1997)					
#	Sex	Haplo.	Pop.	Island	GenBank	Pop.	Island	*q*	Pop.	Island	*L_1_*	Pop.	Island	*L_2_*
CDRS017	M	78	PBR	Isabela	AF548281	FLO	Floreana	0.879	FLO	Floreana	22.20	PBL	Isabela	22.35
CDRS032	M	78	PBR	Isabela	AF548281	FLO	Floreana	0.798	PBL	Isabela	20.45	FLO	Floreana	21.79
CDRS040	F	78	PBR	Isabela	AF548281	FLO	Floreana	0.940	FLO	Floreana	20.63	LT	Isabela	25.40
CDRS042	F	78	PBR	Isabela	AF548281	FLO	Floreana	0.909	FLO	Floreana	18.26	AGO	Santiago	22.66
CDRS043	F	78	PBR	Isabela	AF548281	FLO	Floreana	0.923	FLO	Floreana	23.59	PNT	Pinta	28.14
CDRS044	F	78	PBR	Isabela	AF548281	FLO	Floreana	0.930	FLO	Floreana	22.22	ESP	Española	26.90
***CDRS047***	M	83	PBR	Isabela	AF548286	FLO	Floreana	0.942	FLO	Floreana	21.99	PBR	Isabela	28.66
CDRS106	F	78	PBR	Isabela	AF548281	FLO	Floreana	0.914	FLO	Floreana	22.21	ESP	Española	28.36
CDRS107	F	78	PBR	Isabela	AF548281	FLO	Floreana	0.859	FLO	Floreana	20.42	PBL	Isabela	25.74

Individuals are listed according to the *ex situ* collection in which they currently reside with acronyms as in “Materials and Methods” in the text. Unknown tortoises are assigned to a population of origin based on the location of a shared mtDNA haplotype previously sampled in the wild with corresponding GenBank accession numbers. All individuals possessed one of two “non-native” haplotypes originally sampled on northern Isabela in Puerto Bravo (PBR) (see text for more details). Other population and island locations are specified by acronyms as in Figure 1. Population and island assignment according to the microsatellite genotypic data and the tests of Rannala and Mountain (1997) and Pritchard et al. (2000) are indicated by their corresponding likelihood values (*L_1_* & *L_2_*) and membership coefficients (*q*), respectively. The individual with a “Floreana-like” mtDNA haplotype and congruent nuclear assignment to Floreana is in bold italics.

**Figure 2:**
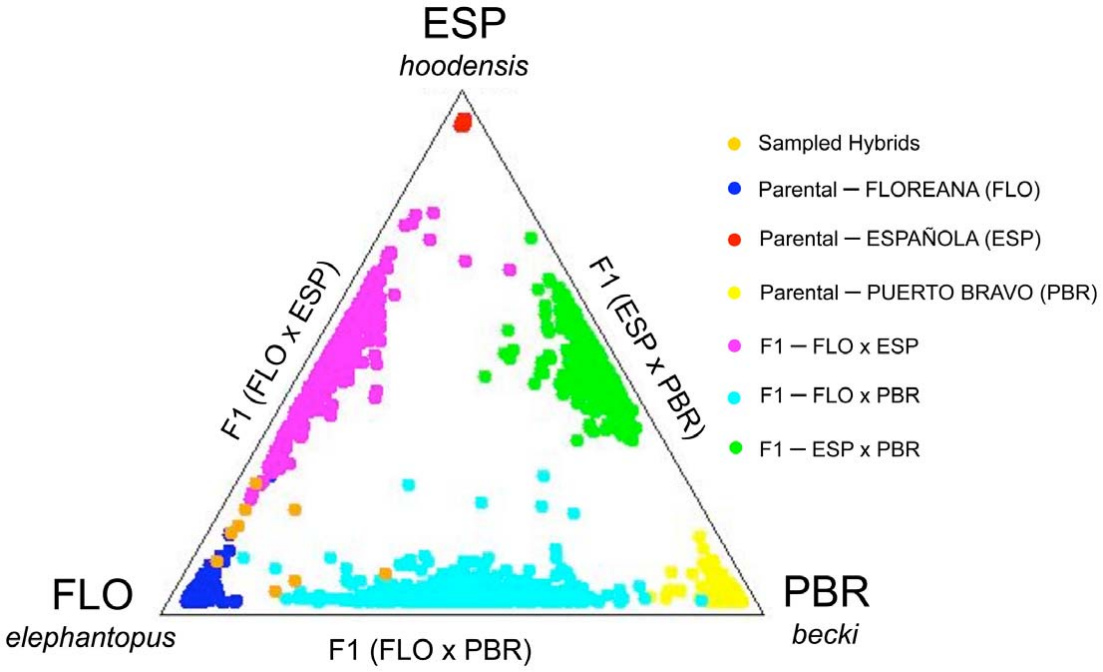
Patterns of Floreana mixed ancestry as revealed by genotype simulations and Bayesian clustering analyses. STRUCTURE triangle plot revealing clustering patterns of individuals of Floreana mixed ancestry with simulated parental and F1 genotypes for all possible pairwise comparisons involving Puerto Bravo (PBR), Floreana (FLO) and Española (ESP) populations. For display purposes, only simulated parental and F1 distributions are shown. Colors according to embedded legend.

The triangle plot in Figure 2 depicts a fine-scale examination of the history of mixed ancestry in the nine captive individuals that assigned to Floreana, obtained through *q*-value distributions of 500 simulated genotypes each of parental, F1 hybrids, F2 hybrids, and B2 and B3 backcrosses for all pairwise comparisons between Puerto Bravo *C. becki* (Isabela), *C. hoodensis* (Española), and *C. elephantopus* (Floreana). One individual (CDRS 40) falls distinctly within the Floreana parental *q*-value distribution, with five others exhibiting strong Floreana ancestry within the Española-Floreana F1 hybrid distribution (Figure 2). Three additional individuals clustered within the Puerto Bravo-Floreana F1 hybrid *q*-value distribution with varying affinities to Floreana. Although these results are clearly indicative of some degree of Floreana ancestry for all nine individuals, additional loci will be necessary to further discriminate between F1 and higher-order hybrids and backcrosses for many of them (Figure 2).

**Figure 3:**
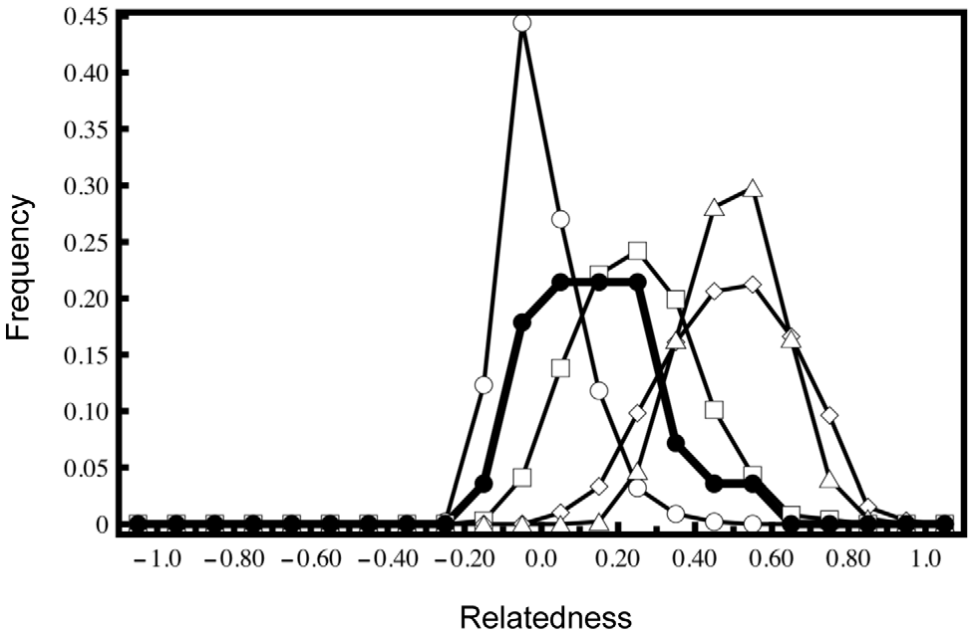
Relatedness structure of captive individuals of Floreana mixed ancestry. Distribution of observed Lynch and Ritland's [36] relatedness values calculated for CDRS individuals of detected Floreana ancestry (•) overlaid upon those calculated from 1000 simulated unrelated (○), half-sibling (□), full-sibling (△), and parent-offspring (◊) dyads.

There is a high degree of relatedness among the CDRS individuals exhibiting signatures of Floreana ancestry [mean pairwise relatedness (*r_xy_*) = 0.15]. Overall, the observed distribution of pairwise relatedness values among the CDRS individuals of Floreana ancestry overlaps substantially with simulated second order (half-sibling) and first-order (full-sibling, parent-offspring) distributions (Figure 3). At the individual level, three of the females (CDRS042-044) housed in the CDRS “parental” pen exhibit pairwise relatedness values consistent with full-sibling relationship (*r_xy_* = 0.40−0.61), while a fourth (CDRS040) appears to be their half-sibling (*r_xy_* = 0.31−0.37). None of the females of Floreana ancestry housed in the CDRS “parental” pen possess genotypic profiles consistent with maternity for any living individuals in the program. Yet, three of them (CDRS042-044) are likely grandmothers, exhibiting *r_xy_* ranging from 0.23–0.31 with at least one individual of Floreana ancestry in the CDRS “progeny” pen. Of particular note, CDRS047, the male with congruent mtDNA and nuclear DNA assignment to Floreana, is the likely half-sibling of CDRS044 co-housed in the “parental” pen, consistent with genotypic pairwise relatedness (*r_xy_* = 0.21) and the discrepancy in mtDNA haplotypes (Table 1).

## Discussion

Broad application of DNA analysis of archival material (e.g. museum specimens) has provided critical spatial and temporal components to ecological, evolutionary, taxonomic and conservation-related research [14]. A particularly powerful application of historical DNA analysis for informing *in situ* conservation has been enabling direct incorporation of extinct taxa in comparative studies with extant forms, whether involving “rediscovery” of presumed extinct species [15], refinement of evolutionary relationships [16] or identification of cryptic diversity [17]. Rarely has historical DNA analysis helped inform *ex situ* species recovery efforts [18] as has been demonstrated here with the identification of the extinct *C. elephantopus* already in captivity.

In the current study, we identified six females and three males of mixed ancestry that exhibited high assignment probabilities to the extinct Floreana species. All of these individuals are currently housed at a single breeding facility on Santa Cruz Island in Galápagos, allowing them to play a critical role as founders of a selective captive breeding program for resurrecting *C. elephantopus* without additional transport or disease transmission concerns. Backcrossing as a species restoration technique has long been considered [19] but rarely implemented, especially in long-lived organisms such as Galápagos tortoises [20]. Although time consuming and resource intensive, there is precedent for successful breeding and repatriation in another species of Galápagos tortoise (*C. hoodensis*) endemic to Española [3]. Since the program's inception in 1975, over 2000 individuals have been repatriated to Española originating from 15 initial founders, assisting in population recovery with demonstrated *in situ* breeding [21], [22]. In addition to the nine captive individuals identified in the current study, a recent field expedition to Vólcan Wolf on northern Isabela in December 2008 sampled and tagged over 1600 individuals in an attempt to identify individuals of pure or mixed Floreana ancestry to further populate a breeding and repatriation program. Additional founders will be important for maintaining the genetic health of a Floreana breeding program given the high degree of relatedness among existing CDRS individuals.

Like the recent rediscovery of the Tasman booby [15], this work generally demonstrates the benefits of integrating historical DNA data with more conventional population genetic approaches for elucidating evolutionary patterns and processes. For example, in the absence of population genetic data from the recently extinct *C. elephantopus*, nine Galápagos tortoise individuals of substantial conservation value were previously misassigned to extant species of varying conservation status [4], [5]. This enhanced ability to collect and analyze genetic data from recently extinct species represents a continued expansion of the conservation biologist's toolbox, in this case within an *ex situ* context, to inform strategies for recovering species diversity.

## Materials and Methods

### Taxonomy

The taxonomy of Galápagos tortoises has changed repeatedly since they were first described formally in 1824 [23]. Pritchard [24] provides a thorough account of the history of Galápagos tortoise taxonomy. Currently, fifteen formally described taxa of Galápagos tortoises are generally recognized, 11 of which are extant and threatened by human activities and introductions of non-native species. These taxa have been described as full species of *Geochelone* [8], [25] as well as subspecies of *Geochelone nigra* [24]. A recent taxonomic revision recognizes all Galápagos tortoise taxa as subspecies of *Chelonoidis nigra*, a genus that now includes all South American tortoise species [26]. Here, we continue to recognize the full species status of many of these taxa [sensu 8] that is most consistent with the overwhelming morphological and molecular evidence [27], [28], but adopting the genus-level revision to *Chelonoidis* [26].

### Sampling

The 156 individuals reanalyzed were originally sampled in Burns et al. [4] and Russello et al. [5], all of which were of unknown ancestry at the time of collection from the following institutions: Caloosahatchee Aviary and Botanical Garden, Florida, USA (CABG; n = 25); Galápagos National Park Service Breeding Facility, Santa Cruz, Galápagos (CDRS; n = 60); mainland Ecuador hotels, universities, zoological and private collections (ECU; n = 29); former Wittmer Collection on Floreana, Galápagos [WCF [formerly FLO in 5]; n = 29]; Prague Zoo, Czech Republic (PRZ; n = 2); San Diego Zoo, USA (SDZ; n = 7); and Zurich Zoo, Switzerland (ZUZ; n = 4). The CDRS sampling includes 58 individuals originally analyzed by Burns et al. [4], 23 of which were sampled from the “parental” pen with the remainder housed in “progeny” pens. Two CDRS females that are currently housed with Lonesome George (CDRS106 & CDRS107) and all other individuals were originally analyzed in Russello et al. [5].

### Mitochondrial DNA Analysis

A 695 base pair fragment of the mtDNA control region was reanalyzed for all 156 captive individuals (see Table S1 for GenBank Accession numbers). Degree of sequence similarity was assessed using stand-alone Basic Local Alignment Search Tool (ftp://ftp.ncbi.nlm.nih.gov/blast/) relative to a database of 119 haplotypes recovered from over 1000 individuals sampled from all extant species of Galápagos tortoises [4], [5], [10], [17], [27], [29] as well as museum specimens from the near extinct *C. abingdoni* from Pinta [13] and the extinct *C. elephantopus* from Floreana [9]. All haplotypes are unique to one of the currently described species with the following exceptions: 1) thirteen haplotypes are shared among two or more southern Isabela taxa [30]; 2) one haplotype is shared between *C. becki* and *C. darwini*; and 3) nine haplotypes that are more closely related to haplotypes sampled in other species on other islands than the populations from which they were sampled in the wild (originally termed “aliens” in [27]). See Ciofi et al. [31] for a comprehensive review of previous studies regarding genetic divergence and phylogenetic distinctiveness among the Galápagos tortoises.

### Microsatellite DNA Analysis

Genotypic data at nine microsatellite loci [GAL45, GAL50, GAL73, GAL75, GAL94, GAL100, GAL127, GAL136, GAL263] previously collected and characterized for all captive individuals [4], [5] as well as for 332 individuals sampled from all extant species [17], [29], [32], the near extinct *C. abingdoni* from Pinta [13] and the extinct *C. elephantopus* from Floreana [9] were reanalyzed in the current study. As new analytical approaches for assessing microsatellite data quality have emerged since much of these data were originally collected, we screened the dataset for null alleles using MICRO-CHECKER [33]. Five out of 153 (i.e. nine loci for 17 populations) comparisons showed evidence of null alleles. Given this very low frequency, data at loci exhibiting null alleles in identified populations were removed prior to population genetic analyses. Following this culling, missing data remained minimal throughout the data set (2.8%).

Captive individuals were assigned to island, species and, in many cases, population based on their multi-locus genotypes using two different approaches. First, the Bayesian model-based clustering method of Pritchard et al. [12] was employed as implemented in Structure 2.3 [34]. Run length was set to 1,000,000 MCMC replicates after a burn-in period of 500,000 using correlated allele frequencies and prior population information following Russello et al. [13]. Membership coefficients (*q*) of the captive individuals in one or more of the reference populations represent the fraction of its sampled genome that has ancestry in that population. In addition, the exclusion-simulation test of the partial Bayesian assignment method of Rannala and Mountain [11] was used to assign individuals to the two closest natural populations where the likelihoods of its genotype occurring were the highest (*L_1_* and *L_2_*) as implemented in GENECLASS [6]. The exclusion threshold was set to 0.01, relative to a distribution estimated from 10,000 randomly generated genotypes.

To evaluate the validity of population assignments and to identify the possible range of *q*-values for potential purebreds and different hybrid classes, a series of simulations were conducted for parental, hybrid, and backcrossed genotypes [13], [35]. Specifically, 500 individuals were simulated for each parental population, as well as for all pairwise combinations of F1 hybrids, F2 hybrids, and B2 and B3 backcrosses. In this case, multi-locus genotypic data collected from population samplings on Floreana Island, Española Island, and Puerto Bravo on Vólcan Wolf on Isabela Island were used as the parental populations for genotype simulations. These simulated datasets were analyzed in STRUCTURE 2.3 [34] using the previously described parameters.

Pairwise relatedness [*r_xy_*; Lynch and Ritland [36]] values were calculated for all CDRS individuals of detected Floreana ancestry using the software iRel [37] implementing the “leave one out” option and using starting allele frequencies based on putatively unrelated individuals in the “parental” pen only. The estimator of Lynch and Ritland [36] was chosen as it has been demonstrated to minimize type II error (ex. full-siblings misclassified as unrelated) relative to other estimators such as Queller and Goodnight [38], an important consideration when using marker-based relatedness within *ex situ* population management programs aimed at avoiding inbreeding [39]. To visualize the distribution of relatedness among the CDRS individuals of detected Floreana ancestry, the frequencies of observed pairwise *r_xy_* estimates for all possible comparisons were plotted with those calculated from simulated distributions of known relatedness (unrelated, half-sibling, full-sibling and parent-offspring) following the approach in Russello and Amato [39]. Specifically, iRel [37] was used to simulate 1000 pairs each of unrelateds, half-siblings, full-siblings and parent-offspring using starting allele frequencies based on putatively unrelated individuals in the “parental” pen only. Lastly, allele transmission patterns were directly examined for CDRS individuals of detected Floreana ancestry to investigate putative maternity and paternity.

## Supporting Information

Table S1 Lineage identification of captive Galápagos tortoises of unknown ancestry based on mtDNA and microsatellite data. Individuals are listed according to the *ex situ* collection in which they currently reside with acronyms as in “Materials and Methods” in the text. Unknown tortoises are assigned to a population of origin based on the location of a shared mtDNA haplotype previously sampled in the wild with corresponding GenBank accession numbers. Individuals possessing a “non-native” haplotype originally sampled on northern Isabela in Puerto Bravo (PBR) or Piedras Blancas (PBL) indicated by *. Other population and island locations are specified by acronyms as in Figure 1. Population and island assignment according to the microsatellite genotypic data and the tests of Rannala and Mountain (1997) and Pritchard et al. (2000) are indicated by their corresponding likelihood values (L1 & L2) and membership coefficients (q), respectively.
(0.08 MB XLS)
